# Bicolor Tuning and Hyper-Reflective Color Switching Based on Two Stacked Cholesteric Liquid Crystal Cells with Asymmetric Electrothermal Optical Responses

**DOI:** 10.3390/molecules29112607

**Published:** 2024-06-01

**Authors:** Hsin-Kai Tseng, Po-Chang Wu, Wei Lee

**Affiliations:** Institute of Imaging and Biomedical Photonics, College of Photonics, National Yang Ming Chiao Tung University, Guiren Dist., Tainan 711010, Taiwan; tonywijaya0199@yahoo.com.tw (H.-K.T.); jackywu0327@gmail.com (P.-C.W.)

**Keywords:** cholesteric liquid crystal, pseudo-dielectric heating, reflection bandgap, hyper-reflective color switching, bicolor tuning

## Abstract

We propose a double-cell cholesteric liquid crystal (CLC) device composed of a left-handed (LH) CLC cell with a pair of sheet electrodes and a right-handed (RH) CLC cell with a tri-electrode configuration characterized by a sheet electrode on the top and an interdigitated electrode on the bottom substrates. Bi-reflected color tuning and hyper-reflective color switching are revealed from this cell stack via the electrothermal control of the central wavelengths of the LH- and RH-bandgaps by voltage-induced pseudo-dielectric heating. The two CLCs are thermally sensitive and exhibit overlapped bandgaps in the field-off state with nearly identical temperature dependence, resulting in a hyper-reflective color at 720 nm at 23.4 °C and 380 nm at 29.8 °C. Upon the application of 4 V_rms_ at 2 MHz across the stacked device to induce pseudo-dielectric heating, two reflective colors can be resolved due to asymmetrical temperature elevations. Accordingly, the difference in wavelength between the two colors increases with increasing voltage through a series cell connection, while maintaining approximately constant via a parallel connection. This study provides a feasible pathway to developing a multifunctional device with electrothermally tunable bi-reflected and hyper-reflective states based on two conventional cell geometries, which is promising for lasers and color-related display applications.

## 1. Introduction

Cholesteric liquid crystals (CLCs) are known to exhibit a photonic bandgap in a given spectral range due to their self-assembled, periodically helical structure along one dimension. This intriguing optical feature, obtained in the Grandjean planar state with a unidirectional helical axis parallel to the light propagating direction, allows a CLC to reflect specific wavelengths of circularly polarized light with the same handedness as the helix. The central wavelength *λ*_c_ and the bandwidth Δ*λ* of a CLC bandgap are characterized by the helical pitch and the ordinary and extraordinary refractive indices of the CLC according to Bragg’s law. Various CLC materials and structures have thus been developed to implement tunable reflective colors in a varying degree of wavelength tunability by modulating the CLC pitch directly through temperature variations [1,2], light irradiation [3,4,5], and applied voltage [6,7,8], or indirectly through electrothermal [9,10,11,12], mechanical [13,14], and hydrodynamic [15] effects. Due to their uniqueness, CLCs have been extensively exploited or introduced for numerous applications, including in color filters [16,17], lasers [18,19], reflective displays [20,21], reflective polarizers [22,23], sensors [24,25,26,27,28], and smart windows [29,30,31]. However, as birefringence in CLCs is typically lower than 0.2 and only one of the two circularly polarized components of light can be reflected, a CLC bandgap is inherently limited to reflect unpolarized light colors within a narrow bandwidth with a reflectance *R*% less than 50%. As such, several approaches toward hyper-reflective colors (i.e., *R*% > 50%) have been suggested in an attempt to meet demands of high reflectivity and/or multicolor reflection for various applications, such as filters, polarizers, and reflectors, among others. One direct way is to stack a left-handed (LH) CLC and a right-handed (RH) CLC with bandgaps at the same wavelengths [32] or to insert a half-waveplate between two identical CLC cells [33]. Radka et al. adopted this double-layer-stacked configuration using two oppositely handed polymer-stabilized CLCs and demonstrated electro-mechanically induced tuning in reflectivity of a hyper-reflection bandgap at a certain wavelength and splitting as well as broadening in bandgaps [34]. Another widely investigated approach is to acquire hyper-reflectivity in a single polymer-stabilized CLC cell by injecting an LH (RH)-CLC into a cell containing an RH (LH) polymer network template based on the wash-out/refill method [35,36,37]. For practical applications, however, the fabrication procedure is relatively complicated, and the resultant bandgap properties can hardly be modulated by an externally applied electric field.

The enclosure of a liquid crystal (LC) between a pair of glass substrates, deposited with transparent indium–tin oxide (ITO) as the conductive layer, is the most commonly used LC cell geometry for electro-optic applications. Since the resistance of ITO is actually non-zero, such a sandwiched configuration is equivalent to a series connection of an LC capacitor and an ITO resistor. As a result, a pseudo-dielectric relaxation with zero capacitance at the high-frequency limit is unavoidably induced in the complex dielectric spectrum. Recently, we have developed an electrothermal approach for low-voltage-driven, full-visible color tuning from a single-handed thermoresponsive CLC with negative dielectric anisotropy based on the pseudo-dielectric heating (PDH) effect [10,11]. Unlike well-known dielectric heating, where the corresponding dielectric relaxation stems from the molecular rotation of the dielectric material used, PDH is attributed to the induction of the pseudo-dielectric relaxation from the cell geometry. We have experimentally and theoretically confirmed that the referred voltage-induced temperature elevation is primarily dominated by the electrode resistivity and the specific heat conductivity of the cell. Inspired by the findings mentioned above [11], the PDH-enabled electrothermal effect has been further applied for color tuning of a thermoresponsive CLC with positive dielectric anisotropy [12] or with a comb electrode [38], and for tristate switching in a tristable smectic A (SmA) LC device [39], and to the formation of uniform lying helix state in a CLC [40].

In this study, a hyper-reflection bandgap, whose central wavelength can be passively tuned by the temperature, was obtained by stacking an LH-CLC and an RH-CLC with the same thermoresponsive properties. Alluring optical characteristics showing electrothermal modulation of bi-reflected and hyper-reflective colors from this stacked structure were demonstrated by fabricating the LH-CLC cell with a pair of sheet-electrode-coated substrates and the RH-CLC cell with a sheet electrode on the top substrate and a pair of comb electrodes (i.e., an interdigitated electrode) on the bottom substrate (to form a tri-electrode structure). The design of the two CLC cells with different electrode configurations aims to elicit asymmetric electrothermal optical responses in the cells by PDH heating under a constant alternating-current (AC) voltage applied across the stacked CLC device. Electrothermo-optical responses of the two bandgaps to the external voltage under series and parallel connections of the two cells were investigated and accounted for by Kirchhoff’s circuit laws.

## 2. Results and Discussion

### 2.1. Passively Thermal and Actively Electrothermal Modulation of the Hyper-Reflective Bandgap

**Figure 1:**
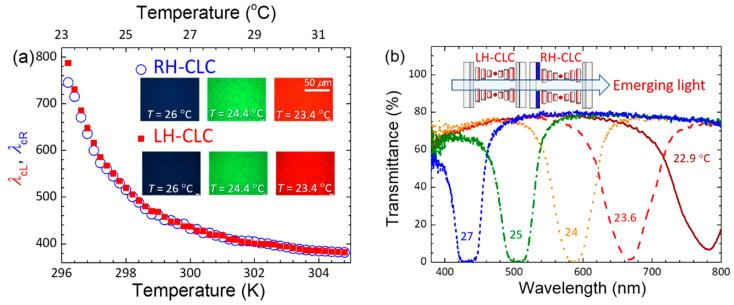
(**a**) Temperature-dependent central wavelengths of the RH-CLC and LH-CLC in the field- off state, and (**b**) transmission spectra of the stacked CLC device at various temperatures. The inset in (**a**) shows *reflective* red, green, and blue images of the individual CLC cells monitored through a POM at three designated temperatures.

The thermoresponsive optical features of the individual RH-CLC and LH-CLC cells, as well as the stacked combination in the field-off state, were evaluated based on the temperature-dependent central wavelength of the RH (*λ*_cR_) and the LH (*λ*_cL_) bandgaps, along with reflective optical textures at given temperatures, as depicted in Figure 1. The temperature as a variable was precisely adjusted using a temperature controller. As indicated in Figure 1a, the two curves, showing gradual blueshifts of *λ*_cR_ and *λ*_cL_ with rising temperature, are overlapped virtually across the entire visible spectrum. Additionally, nearly the same optical appearances of red (at 23.4 °C), green (at 24.4 °C), and dark blue (at 26 °C) textures (observed under a polarizing optical microscope (POM) in the reflection mode) are displayed in the inset. One can see that the fitting parameters of experimental data in Figure 1a with the Keating theory expressed as [41]:

(1) $$\lambda_{c}(T)=\gamma \frac{T_{0}}{T}{\left(1+\frac{\beta }{T-T_{0}}\right)}^{2}$$

where *T* is the absolute temperature in Kelvin (K), *T*_0_ (<*T*) is the SmA–CLC phase transition temperature, and *γ* (in nm) and *β* (in K) are fitting parameters; the fitting parameters of RH-CLC (*γ* = 330.73 nm and *β* = 0.877 K) are very close to those of LH-CLC (*γ* = 330.33 nm and *β* = 0.868 K). The results of such an analysis undoubtedly prove our design by showing that the two oppositely-handed CLCs exhibit comparable optical bandgap responses to the temperature and the same tunable *λ*_c_ at the same temperature. The feasibility of realizing a hyper-reflective bandgap with adaptively adjustable *λ*_c_ in thermal response to the ambient temperature was manifested by transmission spectra as presented in Figure 1b, which shows blue-shifted bandgaps with very low transmittance (implying very high reflectance) at *λ*_c_. One can also see the gradually ascending transmittance at *λ*_c_ from ~0% at *T* = 27 °C to ~7% at *T* = 22.9 °C, suggesting the increasing mismatch level of RH- and LH-bandgaps as the temperature dropped, approaching the CLC-to-SmA phase transition point. The maximal transmittance of *ca.* 80%, rather than 100%, at the wavelengths outside the bandgap might be ascribed to ITO absorption (in the shorter-wavelength regime) and reflections at interfaces.

**Figure 2:**
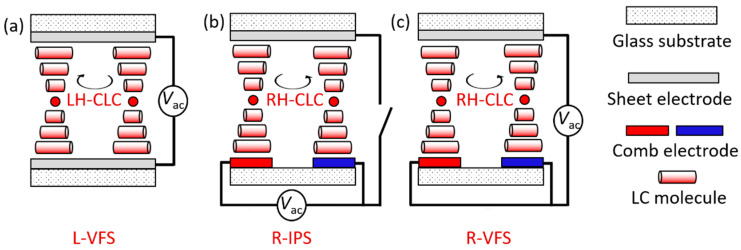
Schematics of cell configurations and electrically driving schemes of (**a**) LH-CLC in the VFS mode with a voltage connection to the top and bottom sheet electrodes, (**b**) RH-CLC in the IPS mode with a voltage connection to the bottom comb electrodes, and (**c**) RH-CLC in the VFS mode with a voltage connection to the top sheet and the common bottom comb electrodes.

**Figure 3:**
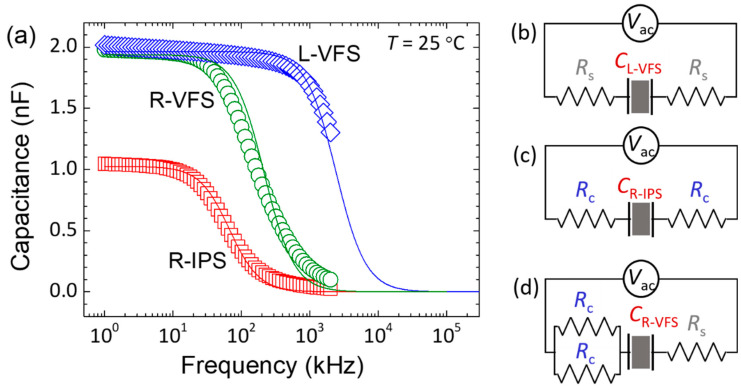
(**a**) Capacitance spectra of the LH-CLC cell in the VFS mode (L-VFS) and the RH-CLC cell in the IPS (R-IPS) and VFS modes (R-VFS), showing *C*_L-VFS_ = 1.964 nF, *C*_R-IPS_ = 1.026 nF, and *C*_R-VFS_ = 1.948 nF at 1 kHz for the three modes. Schematics of the equivalent circuits of (**b**) L-VFS, (**c**) R-IPS, and (**d**) R-VFS. Here, *R*_s_ and *R*_c_ are resistances of the sheet and comb electrodes, respectively. The experimental data shown in (**a**) are acquired with a probe voltage of 0.5 V_rms_ and the solid lines are the fitting curves following Equation (3).

Subsequently, the electrical and electrothermo-optical responses of the two CLC cells were separately demonstrated by applying an external AC voltage in two manners, namely, the vertical-field switching (VFS) mode and the in-plane-switching (IPS) mode, as illustrated in Figure 2. The VFS and IPS modes are characterized by the directions of electrical polarities perpendicular and parallel to the substrate plane, respectively. The LH-CLC in the VFS mode (L-VFS) can be electrically driven by a voltage across the cell with sheet electrodes on the top and bottom substrates (Figure 2a). The RH-CLC with a tri-electrode design is specific, allowing it to operate in the IPS mode (R-IPS) by applying a voltage to the comb electrodes on the bottom substrate (Figure 2b) or in the VFS mode (R-VFS) by shorting comb electrodes and connecting a voltage to the top and bottom substrates (Figure 2c). Figure 3a illustrates the equivalent circuits for the three driving modes and their corresponding capacitance spectra, measured with a probe voltage of 0.5 V_rms_ at a fixed temperature of 25 °C. Typically, a sandwich LC cell can be regarded as a series connection of an effective resistor from ITO electrodes (rendering an effective resistance *R*_ITO_) and a parallel-plate capacitor from the dielectric LC layer (whose capacitance is *C*_LC_) when the electrode material has finite conductivity. In this work, the equivalent series resistor–capacitor circuits for the three above-mentioned driving modes can be represented as follows: two sheet electrode resistors (each contributing *R*_s_) and an LH-CLC capacitor (determining *C*_L-VFS_) for the L-VFS cell (Figure 3b), two comb electrode resistors (each contributing *R*_c_) and an RH-CLC capacitor (yielding *C*_R-IPS_) for the R-IPS cell (Figure 3c), and two parallel connected *R*_c_ to join *R*_s_ and an RH-CLC capacitor (giving *C*_R-VFS_) in the VFS mode in series (Figure 3d). Due to the “dielectric” loss from the *R*_ITO_–*C*_LC_ circuit, a so-called pseudo-dielectric relaxation can be inevitably observed in a specific frequency range in association with the complex dielectric spectrum. The corresponding pseudo-dielectric relaxation frequency *f*_PR_ can be calculated by the following:

(2) $$f_{\mathrm{PR}}=\frac{1}{2\pi R_{\mathrm{ITO}}C_{\mathrm{LC}}}$$

Since both *R*_ITO_ and *C*_LC_ of an LC cell are crucial factors governing the pseudo-dielectric relaxation and the corresponding heating behavior, we strategically opted for comb electrodes with a significantly higher resistivity than that of the sheet electrodes. This choice sought to highlight the LH- and RH-CLC cells with noticeably distinct PDH-dominated electrothermal effects in this study. This conception is initially supported by the capacitance spectra presented in Figure 3a, which reveal diverse capacitance dispersions for the three modes. As evidenced previously, the obtained capacitance spectra in the investigated frequency range (i.e., 1 kHz−2 MHz) in Figure 3a are factually dominated by the pseudo-dielectric relaxation. Here, both space-charge polarization from the transport of mobile ions and orientational polarization from the rotation of LC molecules are excluded because of the use of the nematic host with high resistivity and negative dielectric anisotropy [10,11]. Given that capacitance is proportional to dielectric permittivity, or the real part of the dielectric constant, it is analogous to characterizing the pseudo-dielectric relaxation in terms of the corresponding *f*_PR_, the high-frequency capacitance *C*_H_, and the capacitance in the low-frequency limit, *C*_LC_, by the Debye model [10]:

(3) $$C\left(f\right)=C_{H}+\frac{C_{\mathrm{LC}}-C_{H}}{1+{\left(\frac{f}{f_{\mathrm{PR}}}\right)}^{2}}$$

By letting *C*_H_ be null [42] and fitting the experimental data displayed in Figure 3a with Equation (3), one can deduce *f*_PR_ = 1417.17 kHz for L-VFS, *f*_PR_ = 66.57 kHz for R-IPS, and *f*_PR_ = 151.41 kHz for R-VFS. By substituting *f*_PR_ and *C*_LC_ (static values, say, at 1 kHz) into Equation (2), the estimated values of *R*_ITO_ are 0.056 kΩ = 2*R*_s_ for L-VFS, 2.32 kΩ = 2*R*_c_ for R-IPS, and 0.54 kΩ = 0.5*R*_c_ *+ R*_s_ for R-VFS, supporting our design pertaining to the electrode resistivity, where the resistance of the comb electrode (*R*_c_ ~ 1.16 kΩ) is much greater (or at least 40 times larger) than that of the sheet electrode (*R*_s_ ~ 0.028 kΩ). Note that the three deduced *R*_ITO_ values are in consistence with the results of —0.055 kΩ for L-VFS, 2.3 kΩ for R-IPS, and 0.57 kΩ for R-VFS—directly measured by an LCR meter. Furthermore, the pseudo-dielectric relaxation leading to the capacitance dispersion in each of the three driving modes can be simulated by substituting the relevant parameters into Equation (3) (solid lines in Figure 3a), showing great consistency with the experimental data.

**Figure 4:**
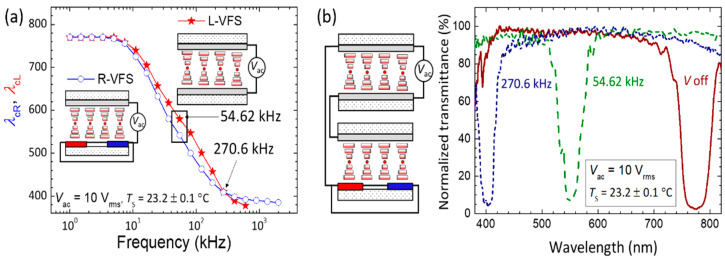
(**a**) Frequency dependence of *λ*_cR_ of R-VFS and *λ*_cL_ of L-VFS driven by 10-V_rms_ applied voltage and (**b**) electrothermally induced hyper-reflective bandgap shift from *λ*_c_ ~ 770 nm (dark-red curve) at *V*_ac_ = 0 V_rms_ to *λ*_c_ ~ 550 nm (green curve) by *V*_ac_ = 10 V_rms_ at *f* = 54.62 kHz, and *λ*_c_ ~ 410 nm (blue curve) by *V*_ac_ = 10 V_rms_ at *f* = 270.6 kHz across the stacked L-VFS/R-VFS device in parallel connection of cell electrodes.

Utilizing PDH to elevate a cell temperature, Figure 4a manifests frequency modulations of *λ*_cL_ in L-VFS and *λ*_cR_ in R-VFS at 10 V_rms_. The surrounding temperature was controlled at *T*_s_ = 23.2 ± 0.1 °C, ensuring that the LH- and RH-bandgaps overlap at *λ*_cL_ ~ *λ*_cR_ ~ 770 nm in the field-off condition. Upon the application of a VFS voltage to the LH-CLC cell (L-VFS), *λ*_cL_ started to blueshift with increasing frequency *f* > 7.39 kHz, reaching ~380 nm at *f* = 602.3 kHz. Further increasing the applied frequency (*f* > 602.3 kHz) gave rise to a blueshift to an ultraviolet wavelength, which cannot be detected by the optical spectrometer used. In contrast, for the R-VFS mode, the onset frequency for modulation of *λ*_cR_ is ~4.95 kHz, and the *f*–*λ*_cR_ plot decreased monotonously with rising frequency, declining slowly beyond *f* = 270.6 kHz and finally reaching a minimal value of *λ*_cR_ = 385 nm at *f* = 2 MHz. The observed PDH-induced bandgap tuning as delineated in Figure 4a can be explained by the frequency-dependent temperature elevation (Δ*T*) in the stationary state [11]:

(4) $$\Delta T=\left[\frac{V^{2}C_{\mathrm{LC}}}{h}\right]\left(\frac{2\pi f^{2}/f_{\mathrm{PR}}}{1+{\left(f/f_{\mathrm{PR}}\right)}^{2}}\right)$$

where *h* is the specific heat conductivity of the cell, attributed to the substrate material. As *f* >> *f*_PR_, Equation (4) can be simplified as follows [43]:

(5) $$\Delta T_{\max}\approx \frac{V^{2}}{hR_{\mathrm{ITO}}}$$

This implies that Δ*T* reaches a saturated maximal value (Δ*T*_max_), becoming independent of the frequency in the condition of *f* >> *f*_PR_. Because *f*_PR_ = 1417.17 kHz of L-VFS is about 10 times higher than that of R-VFS (*f*_PR_ = 151.41 kHz), *λ*_cR_ tuned by the voltage frequency is expected to attain a minimal value at a relatively lower frequency, which is in good agreement with our experimental results (Figure 4a). Notably, Figure 4a reveals that both the *f*–*λ*_cL_ and *f*–*λ*_cR_ curves ensure a tunable range between 770 nm and 410 nm at *f* ≤ 270.6 kHz for a fixed applied voltage of 10 V_rms_. The maximal separation between *λ*_cL_ and *λ*_cR_ is ~37.5 nm at *f* = 54.62 kHz. This suggests the possibility of creating a hyper-reflective bandgap with a tunable *λ*_c_ covering the visible-light regime between 770 nm and 410 nm through the PDH-induced electrothermal effect in a stack of the designated LH-CLC cell and RH-CLC counterpart (in the VFS mode). For example, by connecting the electrodes of the two cells in parallel to apply the same AC voltage in the L-VFS and R-VFS cells, Figure 4(b) demonstrates that the hyper-reflective color can be generated and tuned from dark-red (*λ*_cL_ = *λ*_cR_ = 770 nm) at *V*_ac_ = 0 V_rms_ to green (*λ*_cL_ = 579.5 nm, *λ*_cR_ = 542 nm) by *V*_ac_ = 10 V_rms_ at *f* = 54.62 kHz and to blue (*λ*_cL_ = *λ*_cR_ = 410 nm) by *V*_ac_ = 10 V_rms_ at *f* = 270.6 kHz.

### 2.2. Electrothermal Tunability of Bi-Reflected Color from Staked LH- and RH-Bandgaps

**Figure 5:**
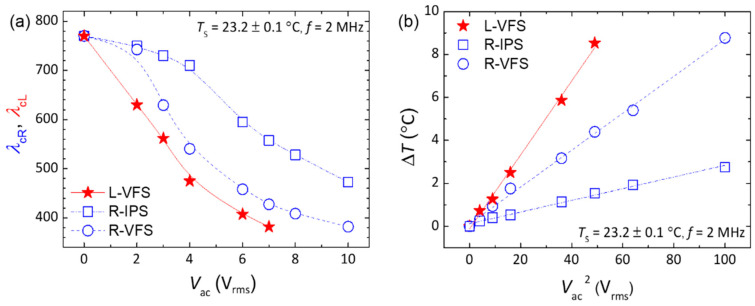
(**a**) Voltage dependence of *λ*_cL_ of L-VFS and *λ*_cR_ of R-IPS and R-VFS; (**b**) linear correlations between Δ*T* and voltage squared (*V*_ac_^2^) as determined by linear regressions fitted to the experimental data, yielding 0.993 ≤ *r*^2^ ≤ 0.997, where *r*^2^ is the coefficient of determination. The frequency of applied voltage is fixed at 2 MHz and the surrounding temperature set at *T* = 23.2 ± 0.1 °C.

As per Equation (5), the variation in Δ*T* exhibits an inverse proportionality to both *h* and *R*_ITO_ of the cell at a given AC voltage. This suggests that the three driving modes of LH- and RH-CLC cells can manifest distinct electrothermal responses. Figure 5a depicts the voltage dependence of tunable *λ*_c_ for the three cases, where *T*_s_ was similarly set at ~23.2 ± 0.1 °C so that *λ*_cL_ ~ *λ*_cR_ ~ 770 nm at *V*_ac_ = 0 V_rms_. Given that Δ*T* increases with increasing frequency, as described by Equation (4), we fixed the frequency at 2 MHz, which corresponds to the measurement limit of the instrument used (LCR meter, Keysight E4980A, Santa Clara, CA, USA). An elevated external voltage prompts a temperature rise in the cell via PDH, subsequently bringing forth a blueshift in *λ*_c_. Figure 5b illustrates the influence of voltage magnitude on Δ*T*, defined as *T* − *T*_s_, where *T*_s_ = 23.2 ± 0.1 °C and *T* denotes the saturated (i.e., steady-state) temperature resulting from the electrothermal effect. Derived from Figure 5a, PDH-evoked Δ*T* (Figure 5b) can be unambiguously calculated by plugging a corresponding *λ*_c_ value and the parameters *γ* and *β* (previously obtained in Section 2.1) into Equation (1). As rendered in Figure 5b, Δ*T* is linearly proportional to the square of *V*_ac_, as prescribed by Equations (4) and (5). At 10 V_rms_, R-IPS and R-VFS warmed up by 2.74 °C and 8.77 °C, respectively. Meanwhile, with only 7 V_rms_ applied, ∆*T* of L-VFS reached 8.53 °C. In comparison with both R-IPS and R-VFS, L-VFS is more energy-efficient owing to the lower *R*_ITO_ of the sheet electrodes, consequently showcasing a more profound electrothermal effect. Hence, by applying Kirchhoff’s circuit laws for the stacked RH- and LH-CLC cells in series or parallel circuits, one can leverage the discrepancy between the *R*_ITO_ values of the two cells. As a consequence, their electrothermo-optical responses can be differentiated, enabling the generation of bi-reflected color across the entire visible-light spectrum.

**Figure 6:**
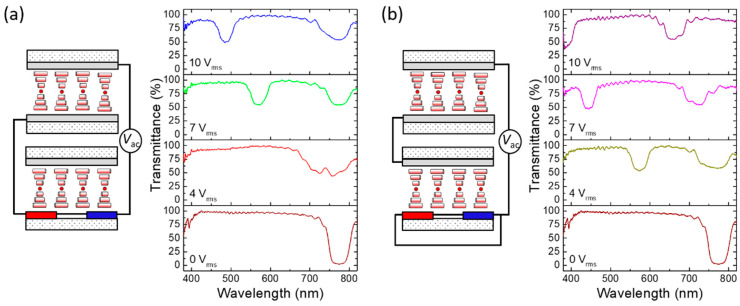
Schematics of the architectures and asymmetric electrothermal optical responses (i.e., normalized transmission spectra) of the stacked LH- and RH-CLC cells connected in series circuits: (**a**) L-VFS + R-IPS and (**b**) L-VFS *+* R-VFS driven by various AC voltages *V*_ac_ at 2 MHz.

By connecting the LH-CLC VFS cell in series or in parallel with the RH-CLC cell under the IPS or VFS driving mode, four sets of the LH- and RH-bandgaps with varying extents of blueshift tuning can be monitored. Figure 6 illustrates the transmission spectra of L-VFS + R-IPS and L-VFS + R-VFS driven by 10-V_rms_ AC voltage at *f* = 2 MHz, with “+” indicating the series connection. To assess the color-tuning capability, we controlled *T*_s_ = 23.2 ± 0.1 °C at *V*_ac_ = 0 V_rms_, ascertaining that the LH- and RH-bandgaps initially coincide. The equivalent circuit featuring the constituting cells (with opposite handedness) satisfies Kirchhoff’s voltage law in the closed loop. According to the voltage divider rule for a series circuit with only two equivalent resistors arising from the CLC cells, voltage *V_i_* divided by each load is proportional to its frequency-independent resistance:

(6) $$V_{i}=\frac{R_{i}}{R_{1}+R_{2}}\cdot V_{\mathrm{ac}}$$

where *R_i_* stands for the individual resistance and *i* = 1, 2. Given that *R*_1_ = 56 Ω for the L-VFS cell and *R*_2_ = 2.3 kΩ for the R-IPS cell, Equation (6) yields the divided voltages of *V*_1_ = 0.024*V*_ac_ and *V*_2_ = 0.98*V*_ac_. As depicted in Figure 6a, when the incremental external voltage at 2 MHz is applied across the stacked L-VFS + R-IPS to induce PDH, the RH bandgap experiences remarkable blueshift due to the asymmetrical temperature rise caused by its much higher divided voltage (for *V*_2_/*V*_1_ = *R*_2_/*R*_1_ > 40). Therefore, when the applied voltage exceeds 4 V_rms_, the blue-shifted RH bandgap can be distinguished from the LH one, and two distinct reflective colors emerge. Even at 10 V_rms_, while the RH bandgap drastically shifts to *λ*_cR_ = 490 nm, a negligible electrothermal heating effect can be implied by the observed LH bandgap remaining virtually unchanged at *λ*_cL_ ~ 770 nm. This is accredited to the low resistance of L-VFS (*R*_s_ ~ 0.028 kΩ), resulting in a very small divided voltage across its sheet electrodes. Comparatively, for the stacked L-VFS + R-VFS device with the composing cells connected in series to produce a resistance ratio (*R*_2_/*R*_1_) of ~10, the divided voltage *V*_2_ = 0.91*V*_ac_ (across R-VFS) is 10 times higher than *V*_1_ = 0.09*V*_ac_ (across L-VFS). Figure 6b sheds light on the voltage drop across the L-VFS cell to be sufficient to induce observable PDH, leading to a more pronounced blueshift of the LH bandgap. Meanwhile, although the divided voltage across R-VFS is lower in comparison with that across R-IPS, the electrothermal effect in R-VFS is stronger than that in R-IPS, enabling superior electrical tunability for *λ*_cR_. As a result, the RH bandgap can blueshift to the lower limit of visible wavelengths (*λ*_cR_ = 395 nm) when the applied voltage is raised up to 10 V_rms_. The results suggest simultaneous and yet asymmetric electrothermal optical responses by virtue of the uneven voltage-induced PDH effects in the LH- and RH-CLC cells.

**Figure 7:**
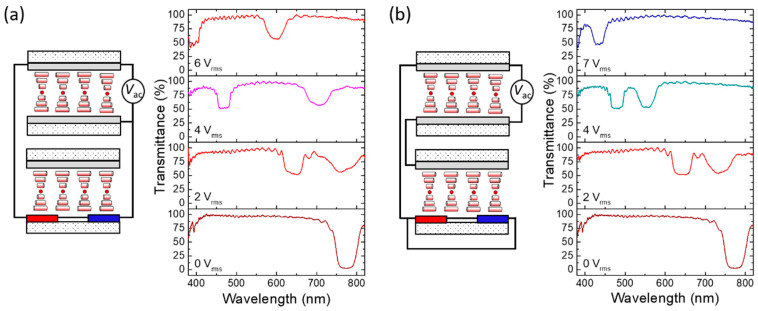
Schematics of the configurations and asymmetric electrothermal optical responses (i.e., normalized transmission spectra) of the stacked CLC cells connected in parallel circuits: (**a**) L-VFS // R-IPS and (**b**) L-VFS // R-VFS driven by various AC voltages *V*_ac_ at 2 MHz.

Figure 7 delineates the transmission spectra of L-VFS // R-IPS and L-VFS // R-VFS driven by various AC voltages *V*_ac_ at *f* = 2 MHz, where “//” denotes the parallel connection. In accordance with the current divider rule for the stacked CLCs connected in parallel, the total current *I* drawn by the circuit is the sum of the currents *I_i_* (*i* = 1, 2) in the two branches of the parallel circuit. One then has the following:

(7) $$V_{\mathrm{ac}}=I\cdot \frac{R_{1}R_{2}}{R_{1}+R_{2}}=V_{i}=I_{i}R_{i}$$

meaning that the voltage drop across a CLC cell is identical to that across the other. The architectural diagrams in Figure 7 reveal that the voltage drop allocated to either branch is equal to the total supply voltage *V*_ac_. The selective reflections characterized by *λ*_cL_ and *λ*_cR_, as shown in both Figure 7a,b, align with the outcomes of the single reflection bandgap depicted in Figure 5a when a specific voltage is applied at *T*_s_ = 23.2 ± 0.1 °C. Drawing from the results in Figure 5b, a notable disparity in PDH effect exists between the L-VFS and R-IPS cells. In consequence, when *V*_ac_ is applied through the parallel connection of the electrodes of the stacked L-VFS/R-IPS device, the LH- and RH-bandgaps exhibit varying degrees of blueshift, as exhibited in Figure 7a. The bicolor reflection bandgaps of the L-VFS // R-IPS configuration generate a variety of mixed colors. For instance, both yellow light (*λ*_cR_ = 598 nm) and violet light (*λ*_cL_ = 392 nm) can be simultaneously reflected at *V*_ac_ = 6 V_rms_ to produce amber color. Moreover, the findings, as seen in Figure 7b, suggest that, as the applied voltage increases to bring about prominent bandgap blueshifts, the spectral separation between the two individual reflection bandgaps of L-VFS // R-VFS narrows compared with that of L-VFS // R-IPS. This narrowing is credited to the more comparable PDH strengths between L-VFS and R-VFS, as evidenced by the results presented in Figure 5b. Therefore, depending on the electrode configuration and resistivity, the designed double-cell CLC device can showcase varying levels of blueshift in the LH- and RH-bandgaps and, hence, a substantial variety of mixed colors.

## 3. Materials and Methods

### 3.1. Materials and Sample Preparations

The LC host MLC6608 (with negative dielectric anisotropy Δ*ε* = −4.2 at *f* = 1 kHz and *T* = 20 °C) and chiral additives S811 (with left-handedness) and R811 (with right-handed chirality) were all purchased from Merck (Darmstadt, Germany) and used as received. Following our previous work [10], 55-wt% MLC6608 was mixed with 45-wt% S811 to obtain a thermoresponsive (i.e., temperature-sensitive) LH-CLC. Its phase sequence is SmA–21 °C–CLC–39 °C–I (isotropic) and *λ*_c_ fully tunable in the visible region by varying temperature. To prepare an RH counterpart with identical phase sequence and temperature-dependent spectral features, R811 as the enantiomer of S811 was incorporated into the nematic MLC6608 at the same concentration (*viz.* 45 wt%).

**Figure 8:**
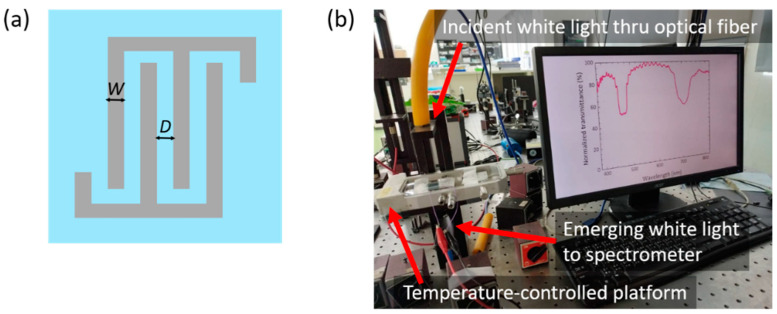
(**a**) Schematic of the interdigitated electrode pattern (top view) with the width *W* = 5 μm and spacing *D* = 5 μm; (**b**) photo showing the setup for transmission spectroscopic measurement of the CLC bandgap.

Two sandwiched cells with different electrode designs were prepared. LH-CLC was injected into a cell consisting of two sheet-ITO-electrode-covered glass substrates. The other cell, filled with RH-CLC, was fabricated with a three-terminal electrode structure by depositing a sheet ITO electrode on the top substrate and an interdigitated ITO electrode array (possessing both the electrode width *W* and spacing *D* of 5 μm as shown in Figure 8a) on the bottom substrate. The inner surfaces of all glass substrates were coated with the aligning agent SE-150 (Nissan Chemical, Tokyo, Japan) to promote homogeneous orientation of LC molecules near the substrates and, thus, the Grandjean planar state in the CLC phase. The thicknesses of the two types of cells were 8 μm, as determined by ball spacers. In the electrothermal experiment, the LH- and RH-CLC cells were stacked in either a series or parallel circuit. They were thermally isolated to prevent any heat exchange between them.

### 3.2. Measurements

All measurements were performed at a fixed surrounding temperature (*T*_s_ = 23.2 ± 0.1 °C) with a temperature controller (Linkam LTS120E, Redhill, UK). Figure 8b demonstrates the instrumentation utilized, which includes a fiber-optic spectrometer (Ocean Optics HR2000 plus, Dunedin, FL, USA) in conjunction with a halogen light source (Ocean Optics HL2000), to investigate the effects of temperature and applied AC voltage on the bandgap properties of CLC cells, in wavelengths spanning from 380 to 820 nm. Capacitance spectra in the frequency regime between 1 kHz and 2 MHz were acquired with a high-precision LCR meter (Agilent E4980A), enabling the exploration of pseudo-dielectric relaxation behaviors. An adjustable voltage, ranging from 0.5 V_rms_ to 10 V_rms_ and oscillating at frequencies between 1 kHz and 2 MHz, was supplied to a CLC cell or two cells connected in a circuit by the same LCR meter. This approach was adopted to eliminate potential systematic errors in voltage conditions that could arise from the use of different instruments.

## 4. Conclusions

We have ultimately presented a color-tunable, multifunctional stacked CLC device with high contrast, taking advantage of the influence of the cell condition on the electrothermal effect stemming from pseudo-dielectric heating. This stacked CLC structure comprises an LH-CLC cell with sheet electrodes and an RH-CLC cell with a tri-electrode configuration. Through electrothermal control, we successfully attained bi-reflected color tuning and hyper-reflective color switching in the *λ*_cL_ and *λ*_cR_ of the LH- and RH-bandgaps. The LH-CLC and RH-CLC utilized are thermally sensitive, designed to display temperature-dependent bandgaps, and engendering nearly identical blueshifts of *λ*_cL_ and *λ*_cR_. By adjusting the surrounding temperature, this stacked CLC device possesses the distinctive capability of tuning the hyper-reflective colors. Our study elucidates that the application of a 2 MHz electric field to the device induces the PDH effects in the oppositely-handed CLCs. Through the series or parallel connection of cell electrodes, one can effectively adjust the two reflection bandgaps simultaneously and manipulate the spectral separation between *λ*_cL_ and *λ*_cR_, depending on the electrode configuration and resistivity. Furthermore, when a 10-V_rms_ voltage is applied to the stacked L-VFS/R-VFS device for the electrodes connected in parallel, the electrothermal response can be modified by altering the voltage frequency to achieve switchable hyper-reflective colors. The unique electrothermal effect specified as PDH has been highlighted by color tuning in this work. Conductive materials for electrodes and electrode patterns can be tailored to optimize the desired electrothermal response. This offers significant application value for multifunctional photonic devices in color rendering and patterning.

## Data Availability

The authors confirm that the data supporting the findings of this study are available within the article.
